# Perspectives on Diversion of Medications From Safer Opioid Supply Programs

**DOI:** 10.1001/jamanetworkopen.2024.51988

**Published:** 2024-12-18

**Authors:** Michelle Olding, Katherine Rudzinski, Rose Schmidt, Gillian Kolla, Danielle German, Andrea Sereda, Carol Strike, Adrian Guta

**Affiliations:** 1 Division of Social and Behavioural Health Sciences, Dalla Lana School of Public Health, University of Toronto, Toronto, Ontario, Canada; 2 School of Social Work, University of Windsor, Windsor, Ontario, Canada; 3 Population Health and Applied Health Sciences, Faculty of Medicine, Memorial University, St John’s, Newfoundland and Labrador, Canada; 4 Department of Health, Behavior, and Society, Bloomberg School of Public Health, Johns Hopkins University, Baltimore, Maryland; 5 London InterCommunity Health Centre, London, Ontario, Canada

## Abstract

**Importance:**

Safer supply programs were implemented in Canada to provide pharmaceutical-grade alternatives to the toxic unregulated drug supply. While research shows clinical benefits and reduced overdose mortality among safer supply patients, medication diversion remains a concern.

**Objective:**

To examine provider (prescribing clinicians and allied health professionals) and patient perspectives on diversion of opioids prescribed in safer supply programs.

**Design, Setting, and Participants:**

In 2021, qualitative interviews and sociodemographic questionnaires were conducted with patients and providers across 4 safer supply programs in Ontario, Canada. Interviews with 21 providers (physicians, nurse practitioners, and allied health professionals) and 52 patients examined experiences implementing safer supply or receiving care. Initial data analysis was conducted from December 2021 to March 2022, and the subanalysis focused on diversion was conducted from December 2023 to March 2024.

**Exposures:**

Participation in safer supply program as a patient or provider.

**Main Outcomes and Measures:**

Data about diversion were coded, extracted, and thematically analyzed.

**Results:**

Of 52 patient participants, 29 (55.8%) were men and 23 (44.2%) were women; 1 was Black (1.9%), 9 (17.3%) were Indigenous, 1 was Latino (1.9%), and 41 (78.8%) were White; and the mean (SD) age was 46.5 (9.6) years. Of 21 provider participants, 6 (28.6%) were men, 13 (61.9%) were women, and 2 (9.5%) were nonbinary; and the mean (SD) age was 37.6 (7.6) years. Participants characterized diversion as a spectrum ranging from no diversion, to occasional medication sharing and loss, to selling all prescribed doses of safer supply (considered rare and easy to detect). Most patients reported they consumed all or most of their prescribed medications and rarely shared or sold their doses. However, providers and patient participants shared that people might share, trade, and/or sell some of their medications with other opioid-using people for multiple reasons. Most prominent reasons for diversion were (1) compassionate sharing with intimate partners and friends to manage withdrawal and overdose risk; (2) selling or trading medications to address their own unmet substance use needs (eg, high opioid tolerance); and (3) medication loss due to poverty, homelessness, and associated vulnerabilities to theft and coercion. Programs used nonpunitive urine drug screening practices and patient self-report to monitor medication use. When diversion was identified, providers described using nonjudgmental conversations to understand patients’ needs and develop mitigation strategies that addressed underlying reasons for diversion, including changing doses and medications prescribed to better match patients’ needs, enrolling eligible intimate partners, and developing safety plans to mitigate vulnerabilities to theft and coercion.

**Conclusions and Relevance:**

Diversion encompasses a wide spectrum of practices (selling, sharing, and loss of medications), and occurs for complex reasons that surveillance and punitive measures are unlikely to mitigate. Diversion may be best addressed by expanding medication options to better match patients’ diverse substance use needs and high tolerance, alongside wraparound social supports.

## Key Points

**Question:**

How and why is opioid diversion occurring in safer supply programs, and how may it be best managed?

**Findings:**

In this qualitative study of 52 patients and 21 providers (prescribing clinicians and allied health professionals), diversion reportedly occurred through compassionate sharing to manage withdrawal and overdose risk, selling or trading medications to address unmet substance use needs, and medication loss due to social vulnerabilities. Programs developed mitigation strategies based on patients’ diverse needs and underlying reasons for diversion.

**Meaning:**

These findings suggest diversion in safer supply programs may be best addressed by expanding medication options to better match patients’ substance use needs, alongside wraparound social supports.

## Teaser

This qualitative study evaluates patient, clinician, and allied health perspectives on diversion of opioids prescribed in safer supply programs in Canada.

## Introduction

North America continues to experience a deadly public health crisis from the toxic and volatile unregulated drug supply. For the past decade, unregulated fentanyl and other highly potent synthetic opioids have been the primary drivers of escalating overdose deaths, accounting for 74 702 deaths in the US in 2023.^1^ However, recent studies indicate the US and Canada are entering a new wave of the drug toxicity crisis. Polysubstance overdose is increasing as drug markets become increasingly unpredictable and characterized by rapid fluctuations in the potency, quality, and composition of substances.^2,3^ Illicitly manufactured opioids are increasingly found to contain unexpected adulterants (eg, etizolam, xylazine, and nitazines) that may elevate the risk of overdose death and long-term adverse health outcomes.^4,5,6^ Jurisdictions have struggled to respond effectively to this complex and rapidly shifting risk environment, with overdose rates remaining high despite scale-up of existing evidence-based interventions such as opioid agonist treatment (OAT), naloxone distribution, drug checking, and overdose prevention sites.^7,8,9^ Novel approaches are urgently needed, including innovations and alternatives to traditional OAT programs, which have well-documented limitations in meeting the withdrawal and risk mitigation needs of people who use fentanyl.^10^

Safer supply is one such intervention that shows promising evidence for preventing overdose and other harms associated with the toxic drug supply. Safer supply is a concept emerging from Canadian harm reduction and drug user liberation movements, and refers broadly to regulated pharmaceutical drugs of known content, quantity, quality, and potency.^11^ Prescribed safer supply, also known as safer opioid supply and prescribed alternatives, has been implemented in Canada under a medical model in which physicians or nurse practitioners prescribe pharmaceutical medications to patients as a safer alternative to the toxic, unregulated drug supply. A small number of Canadian physicians started prescribing safer supply in 2016 to address escalating overdoses among their patients,^12^ with safer supply prescribing later expanded as a risk mitigation response to the concurrent COVID-19 pandemic.^13,14^ Pilot funding from Health Canada starting in 2020 further supported the development and evaluation of dedicated safer supply programs that provide enhanced wraparound health and system navigation supports. In the province of Ontario, safer supply is delivered mostly through primary care clinics and typically includes the prescription of immediate-release opioids (eg, hydromorphone) dispensed daily at a community pharmacy for take-home, unobserved use, often alongside an observed dose of a long-acting opioid such as methadone or slow-release oral morphine (SROM).^15^ An accumulating body of research indicates that safer supply is associated with decreased emergency visits and hospitalization, reductions in overdose and all-cause mortality, and increased self-reported health and well-being.^12,15,16,17,18^

Given these promising findings from Canada, there is growing interest in and calls to implement safer supply in the US and other settings impacted by the drug poisoning epidemic.^10^ Recent US research suggests high acceptability of safer supply among people who use opioids.^19^ Prescription of short-acting opioids for this purpose in the US would require regulatory change,^10^ and there are concerns about opioids being used for nonprescribed purposes.^20^ Diversion, a term that refers to a variety of circumstances in which medications prescribed to 1 person are given, traded, sold, or otherwise taken by someone else, is a concern within public health and medical communities.^21,22^ Diversion is a concern for many pharmaceutical drugs, and prescribed opioid diversion has been documented in chronic pain management and OAT before the advent of safer supply.^19,23,24,25,26,27^ Research documents a wide range of reasons for diversion of opioids prescribed for OAT and pain management, including compassionate sharing, self-treatment of withdrawal, mitigating the volatile unregulated drug supply, and barriers to accessing OAT.^26,28,29,30,31,32,33^ However, there is currently little evidence regarding how diversion is occurring and managed within safer supply programs. Media reporting and other published critiques of safer supply programs center on anecdotal reports that medications are being sold to opioid-naive individuals and becoming a gateway to more powerful unregulated opioids.^21^ However, available research from OAT programs finds that prescribed opioids are diverted primarily to opioid-tolerant individuals, suggesting that diversion from safer supply programs may have potential community benefits from reduced exposure to fentanyl and other toxic adulterants in the unregulated drug supply.^15,34,35,36,37^

Amid controversy and uncertainty about the future of safer supply in Canada, there is an urgent need for evidence regarding diversion within these programs and what it may signify regarding unmet patient and community needs during the drug toxicity overdose crisis.^15,30^ Qualitative and program-level data are required to understand why and how diversion is occurring and how it may be best managed in clinical practice. To address this research gap and inform public health policymaking in the US and Canada, this study draws on qualitative data from an evaluation study of 4 safer supply programs in Ontario, Canada. We explored provider (prescribing clinicians and allied health professionals)– and patient-reported perspectives on medication diversion, including patient practices and programmatic responses.

## Methods

### Study Design and Setting

We conducted a qualitative study about the implementation of 4 prescribed safer supply programs located in Toronto and London, Ontario. Safer supply was delivered in a primary care model, with most patients receiving daily observed doses of long-acting opioids (eg, SROM) at community pharmacies alongside take-home doses of short-acting tablet hydromorphone for unobserved self-administration. One program had a small observed group where medications were ingested onsite for patients considered high risk for overdose due to concurrent heavy alcohol or benzodiazepine use. All programs received funding from Health Canada’s Substance Use and Addictions Program.^17^ Additional details about the programs are provided in the supplementary materials (eTable in Supplement 1).

### Data Generation and Analysis

Between February and October 2021, we conducted semistructured interviews and administered questionnaires to (1) safer supply patients and (2) prescribing clinicians (physicians and nurse practitioners) and allied health professionals (community health workers, case managers, and health system navigators) providing care in safer supply programs, henceforth referred to as providers. Providers were invited to participate by email invitation and through a snowball sampling approach in which we first recruited a census of all prescribers (nurse practitioners and physicians), who in turn referred nurses, community health workers, case managers, and health system navigators within their programs. Clients were recruited through recruitment flyers that were distributed by the research team either directly to clients during site visits, or through providers within participating safer supply programs. We screened clients for eligibility and obtained verbal consent before each interview. Recruitment continued until we reached purposive sampling targets based on the number of clients enrolled in each program. Extra effort was made to include women, people belonging to minoritized racial and ethnic groups, and clients with a history of an HIV or hepatitis C diagnosis. Data reporting followed Consolidated Criteria for Reporting Qualitative Research (COREQ) reporting guidelines.^38^ Research ethics clearance was received from the University of Toronto research ethics board.

Provider questionnaires included items about sociodemographic characteristics, professional roles, years of experience, and training background. Provider interview guides focused on the practical issues and perceptions of the implementation of safer supply programs, processes for enrolling patients, prescribing practices, and other care provided. The provider interview guide included 1 question about policies and practices regarding diversion. Patient questionnaires included items about sociodemographic characteristics, substance use, and medical history. Patient interview guides asked about experiences with safer supply compared with other substance use and health care programs. Race and ethnicity were self-reported by participants using a 2-part question standard for research conducted in Ontario consisting of 1 question about Indigenous identity and 1 question about ethnic origin and race using standardized categories outlined in the Data Standards for the Identification and Monitoring of Systemic Racism.^39^ To protect the identity of providers, we collapsed the race categories into 2 groups (White and minoritized). To mitigate social desirability bias, in the interviews, we first asked participants why they thought other patients might lose, share, or sell safer supply medications. As a follow-up, participants were then asked about their practices and experiences of losing, sharing, or selling medications. Interviews were audio recorded, lasted from 20 to 105 minutes, and were transcribed verbatim. Clients and allied health professionals received $40 for their participation in the interviews, but prescribers were not provided an honorarium. Each site also completed an organizational questionnaire about staffing, enrollment, and services.

### Data Analysis

We used SPSS version 28 (IBM) to manage and analyze questionnaire data, and MAXQDA (VERBI Software) to conduct thematic analysis of interview data. Quantitative data were descriptive and no significance testing was performed. We created codebooks for providers and patients using a priori constructs from the Consolidated Framework for Implementation Research (CFIR), as well as concepts emerging from close reading and discussion of transcripts.^40^ Diversion was included as a subcode under the parent codes and CFIR domains of program context (inner and outer) and implementation process. Each interview was then coded by 1 of 3 primary coders and checked by a second team member to ensure consistency and completeness. Following the Braun and Clarke approach to thematic analysis, 4 team members (M.O., A.G., K.R., and C.S.) exported data related to diversion and met regularly over 3 months to review data, identify key themes, and select illustrative quotes.^41^ Initial data analysis was conducted from December 2021 to March 2022, and the subanalysis focused on diversion was conducted from December 2023 to March 2024.

## Results

We collected data from 52 patients and 21 health care providers across 4 safer supply programs in Ontario, Canada. Of 52 patient participants, 29 (55.8%) were men and 23 (44.2%) were women; 1 was Black (1.9%), 9 (17.3%) were Indigenous, 1 was Latino (1.9%), and 41 (78.8%) were White; and the mean (SD) age was 46.5 (9.6) years. Of 21 provider participants, 6 (28.6%) were men, 13 (61.9%) were women, and 2 (9.5%) were nonbinary; and the mean age (SD) was 37.6 (7.6) years. Sociodemographic characteristics of patients and providers are reported in Table 1 and Table 2, respectively. In our analysis, we identified 3 key themes regarding diversion in safer supply programs: (1) characterizing the spectrum and reasons for diversion practices, (2) programmatic practices to prevent and monitor diversion, and (3) programmatic responses to diversion.

**Table 1.** Patient Participant Sociodemographic Information

Characteristic	Participants, No. (%) (N = 52)
Program site	
London InterCommunity Health Centre	21 (40.4)
Parkdale Queen West Community Health Centre	15 (28.8)
South Riverdale Community Health Centre	11 (21.2)
Street Health	5 (9.6)
Age, mean (SD), y	46.5 (9.6)
Gender^a^	
Man	29 (55.8)
Woman	23 (44.2)
Indigenous^b^	
No	42 (80.8)
First Nation	8 (15.4)
Metis	2 (3.8)
Race and ethnicity^b^	
Black	1 (1.9)
Indigenous	9 (17.3)
Latino	1 (1.9)
White	41 (78.8)
Housing (past 30 d)	
An apartment, house, or condo (rented)	18 (34.6)
Staying with friends, family, or partner	13 (25.0)
Shelters	7 (13.5)
A hotel/motel or room rented by the night, week, or month	6 (11.5)
Homeless^c^	3 (5.8)
An apartment, house, or condo (owned)	2 (3.8)
Supportive or transitional group housing	2 (3.8)
In a long-term care facility	1 (1.9)
Income source (past year)	
Social assistance (ODSP/OW)	51 (98.1)
Paid job	9 (17.3)
Criminalized street economy activities	6 (11.5)
Other government program	5 (9.6)
Sex work	3 (5.8)
Jail or prison	
Ever	45 (86.5)
Past year	3 (5.8)
HIV	
Positive diagnosis	7 (13.5)
Currently on antiretroviral medication	7 (13.5)
Undetectable viral load	7 (13.5)
HCV	
Positive diagnosis (ever)	40 (76.9)
HCV medication	
Yes, finished the treatment	20 (38.5)
No, never	19 (36.5)
Yes, currently on treatment	1 (1.9)
Previous engagement with addiction treatment	
Methadone	45 (86.5)
Buprenorphine	22 (42.3)
Detox	17 (32.7)
Residential treatment	28 (53.8)
Outpatient	17 (32.7)
Treatment in jail	3 (5.8)
Current engagement with OAT^d^	
Yes	12 (23.1)

Abbreviations: HCV, hepatitis C virus; OAT, opioid agonist treatment; ODSP/OW, Ontario Disability Support Program and Ontario Works.

^a^ All clients reported being cisgender.

^b^ Race and ethnicity were gathered using a 2-part question based on guidance provided by the Ontario Government. Participants were asked first if they identify as Indigenous, that is First Nations, Inuit, or Metis, and secondly what race or ethnicity category best describes them. Persons who identified as Indigenous could self-report they were a different race category (eg, White), and participants who did not identify as part of an Indigenous group in Canada could select Indigenous for their race (eg, from an Indigenous group in another country).

^c^ Homelessness was defined as “living on the street, abandoned building, tent/encampment, car/vehicle, outside.”

^d^ Type of OAT was not reported in the sociodemographic questionnaire. Data missing from 2 participants.

**Table 2.** Provider Participant Sociodemographics and Practice History

Characteristic	Participants, No. (%) (N = 21)
Profession	
Nurse practitioner	5 (23.8)
Registered nurse	5 (23.8)
Physician	4 (19.0)
Community health worker	2 (9.5)
Client care support, SOS program administrator	1 (4.8)
Health/systems navigator	2 (9.5)
Outreach worker/site coordinator	1 (4.8)
SOS case manager	1 (4.8)
Site	
Parkdale Queen West Community Health Centre	9 (42.9)
London InterCommunity Health Centre	6 (28.6)
South Riverdale Community Health Centre	3 (14.3)
Street Health	3 (14.3)
Age, mean (SD), y^a^	37.6 (7.6)
Gender^b^	
Woman	13 (61.9)
Man	6 (28.6)
Nonbinary	2 (9.5)
Race and ethnicity	
White	15 (71.4)
Minoritized	6 (28.6)
Time worked at site	
<6 mo	1 (4.8)
6-11 mo	5 (23.8)
1-2 y	3 (14.3)
3-5 y	5 (23.8)
6-10 y	6 (28.6)
>10 y	1 (4.8)
Years worked with people with HIV	
1-2	1 (4.8)
3-5	4 (19.0)
5-10	1 (4.8)
6-10	9 (42.9)
>10	6 (28.6)
Years worked with people who use drugs	
1-2	1 (4.8)
3-5	3 (14.3)
5-10	1 (4.8)
6-10	10 (47.6)
>10	6 (28.6)
Hours worked (per week at SSP)	
20-34	2 (9.5)
35-39	10 (47.6)
40-44	5 (23.8)
45-49	2 (9.5)
>55	2 (9.5)
No. of continuing education courses on substance use or harm reduction	
0	3 (14.3)
1	3 (14.3)
2	6 (28.6)
3	7 (33.3)
5	2 (9.5)

^a^ One practitioner did not provide their age.

^b^ All men and women reported being cisgender.

### Characterizing the Spectrum and Reasons for Diversion Practices in Safer Supply Programs

Providers and patients characterized diversion along a spectrum from no diversion, to occasional sharing of medications, to selling all prescribed doses of safer supply. Completely selling all prescribed daily doses of short-acting tablet hydromorphone was considered a very rare form of diversion by both patients and providers. Most patients reported that they consumed all or most of their prescribed medications and rarely shared or sold their doses. However, patients reported they were aware that others in the program might share, trade, and/or sell some of their medications with other people who used drugs for multiple reasons (Table 3). Both patients and providers described diversion as occurring for compassionate reasons, most often to help intimate partners or friends who used opioids and were experiencing withdrawal and/or were at high risk of overdose from the use of unregulated fentanyl. Providers acknowledged that the levels of hydromorphone they prescribed did not always meet the high opioid tolerance of patients, particularly during the titration period, which also could result in diversion. For example, providers and patients highlighted scenarios of patients selling or trading a portion of their medication for some unregulated fentanyl to close the gap and manage withdrawal symptoms during titration. Providers also reported that some patients sold medications to meet basic survival needs due to poverty (eg, in exchange for a place to stay or food). However, patients emphasized that this was not a desirable way to generate income, given the importance of prescribed medications to their well-being, the low street value of hydromorphone in their community, and concerns that selling medications might jeopardize the sustainability of safer supply programs for themselves and others. A few participants reported instances of patients having medications stolen, being coerced to give away medications, or losing medications. Patients and providers attributed these instances of medication loss to vulnerabilities stemming from housing insecurity, frequent displacement from housing and/or encampments, and lack of secure spaces to store medications.

**Table 3.** Participant Quotations Regarding Reasons for Diversion

Reason	Quotation
Sharing medications with opioid-tolerant partners, family members, and friends to manage overdose risk and withdrawal	One of the situations where I think I have heard [about sharing medications] is in relationships where there’s one person on the program and the other one is not. And so there’s this urgency to “can you help me get my partner on this program because, if not, I feel guilty that I have access and they don’t, and then I feel pressured to share my medication and therefore then I have to continue using fentanyl because it’s not enough for both of us.” (Allied health provider) We know clients want to help people and they see someone who’s going through withdrawal and dope-sick, they want to be able to help their friends, and we know that that probably happens sometimes, where people share medication occasionally with people who are going through it, and they don’t want to see that happen to someone because they know what that feels like [...]. Or we’ve had the clients tell us, “My partner is consistently overdosing on fentanyl, so I’ve been sharing my Dilaudid with them because I don’t want that to happen. (Registered nurse) My ex, he’s now on the program, but when I got on the program, we were living together, we still had our daughter and stuff. But there wasn’t room for him on the program. So I would share [my medication] with him because he was also an addict. (Patient, woman, 30s) I’ve definitely helped somebody out that’s dope-sick, hell yeah, because I would never wanna see somebody like that, and if I have the means to, I’m gonna help ‘em out. I’ve absolutely given up a pill. (Patient, man, 30s) I know people like to argue and say “oh, what if those pills are endin’ up in the wrong hands?” “What if people are selling their pills?” ‘Cause that’s that’s the only argument they have, when it comes to safe supply. “What if people are diverting their pills?” Well, when they’re diverting their pills, the person that’s buying it on the other end is a heavy opiate user, so, end of the day, say that wasn’t there, if the pills, the diverting wasn’t there, they eventually would have ended up doing street fentanyl. So, they’re delaying somebody from doing that. (Patient, man, 20s)
Trading or selling medications to address unmet substance use needs	Yeah. I think [diversion] is just a part of what’s going to happen because folks are now so dependent on that fentanyl, which is why we see our doses have to go up and up to just combat that because of how high the tolerance is getting, because of what’s on the street. So, people are still having to either substitute with small amounts of fentanyl or whatever they’re needing to take in order to keep them how they need to be. (Allied health provider) It’s really strong shit, that fentanyl. Even with methadone, a high dose, it cuts through. I don’t think there’s anything out there that is ready for what fentanyl did. There’s nothing—methadone was there for heroin, but there’s nothing there for fentanyl, that I came across. So it was very hard to close that gap where you’re doing your Dilaudids [hydromorphone]. If you’re doin’ fentanyl all the time, you can’t [expletive] do that. (Patient, man, 40s) I know some people do it [sell medications] because they are addicted to the fentanyl, so they will sell their pills to get the money to buy the fentanyl […]. I’m trying not to. Maybe a couple of days a week. I just can’t get enough [opioids] to get what I need through the day, so I’ll trade a couple of them [medications] or sell a couple of them. (Patient, woman, 30s)
Selling medications to meet basic survival needs	My first reasons to get on it [safer supply] was not the reason why I’m on it now. My first reason to get on it was to sell pills, to make money. And fortunately, that’s not what happened. It saved my life, I could say. I’ve used the pills to inject and it got me off fentanyl for four months. (Patient, woman, 50s) Maybe I could see people doing it [selling medications] to get a pack of cigarettes or something, but I couldn’t see anybody selling their whole medication. That’s just crazy. I wouldn’t be able to do it. (Patient, woman, 50s) I’ve been approached twice [to sell medications] by people who know me, but it [the reason to sell] would have to be financial, right? Somebody who’s on Ontario Disability Support Program [might sell]. ODSP you don’t get much, welfare you’re getting less, right? So right now I’m lucky because, not only am I on ODSP and that, but I’m also working part-time, right? (Patient, man, 50s)
Loss of medications due to displacement, theft, or coercion	Theft is an issue. We’ve had incidences where our patients have been targeted by others in the community, either for personal gain or to steal their drug supply outright. (Allied health provider) Probably homeless people [lose their medications]. ‘Cause they have to have money in their pocket every day, to eat, but they get robbed and stuff, too, they’re robbin’ one another and things like that. Nobody’s ever stolen my meds. I put them in a safe. So I don’t have to worry about it. But they don’t have that out here, right? And so I imagine it’s probably one of the reasons why that stuff happens, right? (Patient, woman, 50s) To lose a dose, maybe they’re on the nod and they get pickpocketed or they drop their dope when they’re not knowingly [doing so]—if they’re homeless. (Patient, man, 30s)

### Programmatic Practices to Prevent and Monitor Diversion

Safer supply programs employed multiple strategies to prevent and monitor diversion (Table 4). Providers described having foundational, open, and nonjudgmental discussions with patients at intake about the potential reasons, risks, and programmatic consequences of diversion. Providers emphasized the importance of maintaining open communication with patients to proactively mitigate potential drivers of diversion, including medication and dosage adjustments to minimize withdrawal. All programs used urine drug screening (UDS) as part of routine care, including when medication changes were requested and to confirm the presence of hydromorphone. However, UDS was not used to identify the use of unregulated drugs and impose penalties. Rather, providers framed UDS as a tool to help patients understand what drugs they had been exposed to in the unregulated drug supply. Patients understood and largely agreed with this use of UDS, which they framed as less invasive and more acceptable than their experiences with UDS in other health care settings, such as methadone programs. Safer supply programs occasionally received reports of diversion from pharmacists, patients’ family members, community members, and other patients. While providers described diligently addressing credible reports of diversion, they expressed concern about the surveillance of patients for reasons discussed in the subsequent section.

**Table 4.** Participant Quotations Regarding Programmatic Practices to Prevent and Monitor Diversion

Reason	Quotation
Establishing expectations about diversion at intake	[At intake] I just let patients know that “when you meet with the doctor, she’s going to be talking about diversion.” We know that it happens. I tell them that we know that it happens, that we try and deter it, and that there are consequences for diverting. Those consequences are discussed with the doctor, and she’ll go into depth with it. I just ask them to be a hundred and ten percent honest and that they’re not going to get in trouble when they’re honest. To just be honest, lay their cards on the table. They take it well. (Allied health provider) Part of the [patient] contract is the rights, responsibilities, it’s about that issue [diversion], and asking for transparency and asking for clients to be honest with us, in the understanding that diversion could happen for a number of reasons and, if it comes up that they’re sharing with somebody because they can’t get on safe supply, we encourage them to talk to us about that so we can connect with that person, and try to see if there’s something we do that way. (Registered nurse) [At intake] we’ll talk about diversion, risks around diversion. We will also have a more honest conversation around recognizing that people sometimes share drugs, whether they’re prescribed or otherwise with loved ones or peers in the community, especially if they see them being really, really sick. And we usually bring that up as, “if you need to do it one time here, that’s not something that I would penalize you for. I would just want to know.” (Physician)
Urine drug screening	Honestly, we’re pretty understanding folks, so it is rare, the cases that we have had deal with a lot of diversion. With some folks strictly selling all of their medications. Which tends to become evident because when folks check in for their appointments, we do urine toxs, so we can see. Everyone tends to think that we’re looking to see what’s in their urine, saying, “Oh, are you using this, that and the other thing?” but we’re actually checking to make sure that their meds are in their urine, so that we know that there’s no diversion happening. (Allied health provider) We’re clear with people that we’re also checking [urine] to ensure that Dilaudid [hydromorphone] is there as well as morphine or whatever other medication we might be prescribing. And we’ll let people know clearly that the only time they would be negatively penalized for what’s in their urine drug screen is Dilaudid is not showing up. (Physician) Once in a while, I do a urine test, to go in to see the doctor, I guess. But, I know a lot of people who don’t agree with the program are worried about diversion so I guess they want to make sure that you’re still taking them. (Patient, woman, 30s)
Investigating reports about diversion	If we’re receiving reports from different people, whether that’s other clients in the program or from a pharmacy that a client is potentially diverting their meds, the prescriber usually has a conversation with them about it. (Registered nurse) We do often get reports of other clients or people calling in saying, “Hey, so-and-so’s selling their meds” in which case we try to do as best of an investigation on it as we can. That one is over my scope of practice and I leave that to the outreach workers and the doctor. (Allied health provider) I’ve known people that have had family members call in and say, “My daughter is selling her pills to get fetty [fentanyl]” and the doctor will either cut them off or give them a warning, and they’ll be under investigation and on probation. And if they find more evidence that they’re selling it, like standing in behind the pharmacy and selling their pills, then they’ll get cut off [from the medications]. (Patient, woman, 40s)

### Programmatic Responses to Diversion

When diversion of medications was identified, programmatic responses varied depending on the extent and underlying reasons for diversion (Table 5). Providers described having honest conversations with patients as a starting point to understand the underlying circumstances that led to diversion and to develop individualized responses. In cases where patients were sharing medications with an opioid-tolerant intimate partner experiencing withdrawal or frequent overdose, some providers prioritized enrolling the partner, so patients were not compelled to share medications. In cases of diversion from loss or theft, safer supply providers described working collaboratively with patients to develop safety plans such as providing lockboxes, observed dosing options, and arranging delivery or secure transport to and/or from pharmacies. If these measures were unsuccessful, prescribers sometimes temporarily restricted take-home doses or required more frequent appointments and UDS until concerns about diversion were addressed. However, prescribers expressed concerns that heightened surveillance through observed dosing or frequent UDS could adversely impact therapeutic relationships and patient engagement with primary care. Prescribers argued these decisions should be made cautiously and on a case-by-case basis, considering the relative benefits and risks of heightened surveillance. When these options were exhausted—or in rare cases where patients were found to be selling all doses of short-acting tablet hydromorphone—providers reported ceasing take-home doses of medications, typically by transitioning patients to a prescription for observed OAT. There were very few accounts of discharges, as providers emphasized the importance of maintaining strong therapeutic relationships with patients who had complex health issues and vulnerabilities both related to and beyond their substance use.

**Table 5.** Participant Quotations Regarding Programmatic Responses to Diversion

Reason	Quotation
Having honest conversations about diversion	We talk to our patients a lot about diversion, about how they have a responsibility to themselves, the community and the integrity of the program. We talk to them a lot about the fact that, if everybody just sells their pills for fentanyl, this program will no longer exist, and we can help nobody. I think, at least in those conversations, my patients seem to understand. (Physician) I don’t know if [diversion] would necessarily result in a discharge so much as a conversation with the person about “what are you getting from the program? What’s going on?” and trying to come up with another strategy. (Nurse Practitioner) I think my doctor is a great lady. And I’m pretty sure she thinks I’m a great lady. I share a lot of things with her. I don’t lie to her. I’m very truthful with her about everything. I have nothing to hide to my doctor. If something is going on, I would let her know. If she was to ask me certain questions, I respond to her very honestly. (Patient, woman, 40s)
Restricting or removing take-home doses	Then there’s more wholesale diversion. I referred to the two women who we had objective evidence that they weren’t using their script at all, they were dumping it for fentanyl or other income. When we have knowledge of that, we remove people from take-home doses because that’s a different kind of diversion. You’re not improving your own health and wellness just by dumping your entire prescription. (Physician) There’s one particular patient, who’s not a patient of mine, but someone who there was a pretty significant concern for diversion for them, and they were given the option to either have their doses observed and this person was moved to observed doses in order to facilitate our comfort level with continuing to prescribe safe opiate supply to them. (Physician) Sometimes when people are very vague and they continue to be very vague about their [medication and drug] use, then sometimes they might switch those people to the observed doses for a while, just to get a sense of how they’re using. (Registered nurse)
Enrolling eligible partners, friends, and family members into safer supply program	We also let people know, if you feel that you’re having to share with the same person a lot I’d rather you do really well in the program and we’d rather you just actually let us know and refer that person to the program and see if we can support them as well. (Physician) Sometimes we have expedited people’s partners into the program […] we’ve had the clients tell us, “My partner is consistently overdosing on fentanyl, so I’ve been sharing my Dilaudid with them because I don’t want that to happen.” So [enrolling the partner] is one way that we try to address diversion. (Registered nurse) I mean, I have shared them [hydromorphone] with my boyfriend a couple of times. My doctor knows that. But, other than the selling and that [sharing with my boyfriend], no, I need them. I need every single one I get […]. So my doctor put him on the priority—we did an intake with him a few weeks ago so now we’re just waiting back to see when we can get him started. (Patient, woman, 20s)
Developing safety plans	We talk to patients pretty regularly about safety planning and how they’re feeling about their safety and their vulnerability when they’re out there on the street. So yeah, we’re doing it pretty regularly and monitoring what we see. We’ll sometimes, as outreach workers, we’re out there in the community, we hear what’s going on, we try and mitigate those situations and, like I said, develop safety plans, provide people transportation in and out of their pharmacy, so bus tickets, cab vouchers, that kind of thing, to try and improve safety. (Allied health provider) But we would have to discuss [unintentional loss of medications] as a team, I believe. Bring it to our clinical meeting and discuss it as a team, if it’s been more than twice. But, yeah, after two times, we’d have to encourage patients to protect their medication better, switch them to observed doses, help them get a lock box, things like that. (Allied health provider)
Discharging patient from safer supply program	There was another woman who was caught on camera not just once, but multiple times, selling her entire script for fentanyl at our consumption site, and so—again, we didn’t discharge her on the first time. We didn’t discharge her on the second time. But since she wasn’t able to stop that behavior, we had to let her go. (Physician) Yeah, they [doctors] are always “don’t do it [divert], don’t do it, and if we catch you, that’s the one way you can get cut off.” You can come in here and pee dirty, where what I mean by peeing dirty is there could be coke in your pee, there could be fetty [fentanyl] in your pee, there could be whatever in your pee. But you won’t get off [the medications]. But you *will* get cut off if they catch you selling your pills. (Patient, woman, 40s)

## Discussion

In our sample of 4 Ontario safer supply programs, patient and provider participants characterized diversion as a spectrum from no diversion, to occasional sharing of medications with others who were opioid-tolerant, to the selling of all prescribed medications—a practice described as rare and easily detected through urine drug screening. Most accounts of diversion fell in the middle, as patients described infrequent sharing, trading, or selling medications to other opioid-using people in their networks for compassionate reasons and/or to address their own unmet substance use needs. Programs conducted routine UDS to confirm the consumption of prescribed medications, a practice that was considered acceptable to most patients in this study given that they were not punished (eg, having doses reduced or being discharged from program) if other substance use was detected. Programmatic responses to diversion depended on the individual patient and reasons for diversion, including enrolling eligible opioid-using partners, creating safety plans, increasing UDS and observed dosing, and adjusting doses to better meet patients’ opioid tolerance.

This study provides a more nuanced account of safer supply diversion than has been depicted in media accounts, statements from politicians, and medical commentaries, which tend to frame safer supply diversion narrowly as the selling of medications to opioid-naive populations such as youths.^21^ Similar to research on OAT,^26,28,29^ patients in our study reported sharing, selling, or losing some doses of their safer supply medications, but emphasized that these medications were diverted to other opioid-using and opioid-tolerant people in their communities (particularly intimate partners). For many patients, many people in their communities had not been able to access safer supply and therefore remained at high risk of overdose from using unregulated fentanyl. This is reminiscent of motives for diversion among pain management patients, which commonly occurs to support self-medicated pain among family and friends.^42^ These compassionate reasons for diversion could support a potential secondary benefit of safer supply in reducing community-level overdose risk, as nonenrolled opioid-tolerant individuals use diverted medications to reduce their exposure to and overdose risk from the toxic, unregulated drug supply.^30,34^

Our data further indicate that safer supply patients were highly motivated to use medications as prescribed when their withdrawal and basic survival needs were met. Consistent with previous research on diversion in OAT programs,^37^ some patients in this study reported selling or trading medications to buy fentanyl to get through the titration period or manage ongoing withdrawal due to high opioid tolerance. As Gagnon and colleagues^16^ report, safer supply diversion is less likely as patients stabilized on the program. Selling doses could be a survival strategy for some patients living in poverty.^35^ However, most patients in this study denied doing so and cited the low resale value of hydromorphone and the importance of safer supply medications to their well-being. While patients and providers expressed concerns about diversion, they felt that discharging patients from care was inappropriate. Participants emphasized that patients would be significantly vulnerable to overdose death and other poor health outcomes if they lost access to the medications, primary care, and wraparound supports provided by safer supply programs. Providers argued that expanded surveillance and punitive responses would likely result in program disengagement, as has been reported in methadone programs.^43^ Instead, they emphasized nonjudgmental conversations with patients as a more successful approach for understanding, preventing, and addressing the underlying drivers of diversion.

This study identifies promising strategies for monitoring and mitigating diversion, many of which are similar to those recommended to address similar diversion motives for opioids prescribed for pain management.^44^ These findings further underscore the need for broader systems changes and clinical innovations to address the complex pressures patients face to divert medications. To address diversion due to unmet opioid tolerance, there is an urgent need to expand safer supply medication options to include medications that better match the potency of unregulated fentanyl, including multiple formulations of prescription fentanyl (ie, patches, tablets, and injectables).^45^ Programs frequently prioritize the admission of eligible opioid-tolerant partners and friends to address compassionate sharing. However, this is not always possible due to program capacity limits (eg, funding and staffing constraints).

Thus, it is necessary to increase the capacity of individual programs through targeted funding and sector-wide investments of sustained funding to offer a safer supply beyond the small number of pilot programs with limited enrollment. More complex and multisectoral approaches are also needed to address diversion via loss, theft, and coercion. Risk mitigation strategies to address this diversion include providing secure spaces for patients to store medications (eg, lockboxes), expanding pharmacy options for safer supply patients, providing home delivery of medications, and arranging patient transportation to and from pharmacies.^46^ However, wraparound supports that connect patients to housing and income supports may ultimately be most critical to mitigating diversion, given the prominence of housing and income insecurity within patients’ reported reasons for selling, sharing, or losing medications.

### Limitations

A limitation facing this and other research is that patients may be uncomfortable disclosing their experiences with or engagement in diversion and emphasize more socially acceptable forms of diversion such as compassionate sharing. To mitigate social desirability bias during interviews, participants were informed that the interviews were being conducted by an external team and assured that data would not be shared with providers. The interview team refrained from using the term diversion itself, which can have negative connotations; instead, we asked patients to describe reasons why medications may be lost, shared, or stolen before asking directly about their practices. When patients described diversion, interviewers probed to understand these circumstances. Our findings underscore the need for additional research to quantify the extent to which diversion occurs and its impacts within the broader community, including unintended harms and potential benefits within the context of a volatile and toxic unregulated drug supply.^35^ Future research should explore the perspectives of those purchasing diverted medications to better understand their needs.

## Conclusions

In this qualitative study of 4 safer supply programs in Ontario, patients and prescribers characterized diversion as a spectrum of practices in which selling all prescribed doses was rare, and no or occasional medication sharing was most common. Diversion reportedly occurred for multiple reasons, most prominently compassionate sharing, unmet substance use needs, and loss of medications due to social vulnerabilities. Expanded surveillance and punitive measures are unlikely to mitigate these types of diversion and may deter the most vulnerable patients from accessing this lifesaving intervention. Our findings underscore the importance of developing and evaluating multifaceted strategies to address the diverse spectrum of medication loss, selling, and sharing practices that may constitute diversion. These strategies should be grounded in respect for patient autonomy and rights to humane health care. Clinical responsibility must be balanced to mitigate the potential harms of diversion with the now well-evidenced health and social benefits of safer supply.

## Notes

Supplement 1. **eTable. **Safer Supply Program Characteristics in February and March, 2021

Supplement 2.
Data Sharing Statement
