# Supplementary material for: Perspectives on Diversion of Medications From Safer Opioid Supply Programs
Source: JAMA Netw Open. 2024 Dec 18;7(12):e2451988. doi: 10.1001/jamanetworkopen.2024.51988 (PMC11656262; doi:10.1001/jamanetworkopen.2024.51988)
Supplement: Supplement 1. — eTable. Safer Supply Program Characteristics in February and March, 2021 [file jamanetwopen-e2451988-s001.pdf]

Supplemental Online Content

Olding M, Rudzinksi K, Schmidt R, et al. Patient and clincian perspectives on diversion of medications from safer opioid supply programs. *JAMA Netw Open*. 2024;7(12):e2451988. doi:10.1001/jamanetworkopen.2024.51988

eTable. Safer Supply Program Characteristics in February and March, 2021

This supplemental material has been provided by the authors to give readers additional information about their work.

© 2024 Olding M et al. *JAMA Network Open.*

eTable. Safer Supply Program Characteristics in February and March, 2021

		London InterCommunity Health Centre	Street Health	South Riverdale Community Health Centre	Parkdale Queen West Community Health Centre (Both sites)
**City**		London	Toronto	Toronto	Toronto
**Service Delivery Location**		Community health centre	Non-profit, community-based organization	Community health centre	Community health centre
**Total number of patients enrolled** (at time of survey)		247	31	46	92
**Number of discharges/ dropouts** (past 6 months)		2	3	25	13
**Prescribers**					
	Total	2	1	1	6
	Physician	1	0	0	4
	Nurse Practitioner	1	1	1	2
**Allied Health**					
	Total	10	2	2	8
	Nurse[a]	3	1	1	4[a]
	Community Health Worker	0	1	1	0
	Care Facilitator/ Case manager	2	0	0	2
	System navigator	1	0	0	2
	Outreach/ in reach worker	3	0	0	0
	Lab technician	1	0	0	0

a. Including 2 registered nurses and 2 licensed practical nurses

© 2024 Olding M et al. *JAMA Network Open.*
