# Supplementary material for: Perspectives on Diversion of Medications From Safer Opioid Supply Programs
Source: JAMA Netw Open. 2024 Dec 18;7(12):e2451988. doi: 10.1001/jamanetworkopen.2024.51988 (PMC11656262; doi:10.1001/jamanetworkopen.2024.51988)
Supplement: Supplement 2. — Data Sharing Statement [file jamanetwopen-e2451988-s002.pdf]

Data Sharing Statement

Olding. Patient and Clinician Perspectives on Diversion of Medications From Safer Opioid Supply Programs. *JAMA Netw Open*. Published December 18, 2024.
doi:10.1001/jamanetworkopen.2024.51988

Data
Data available: No

Additional Information
Explanation for why data not available: This study is based on qualitative interviews which include identifying information to the topic matter.
